# Conditioned pain modulation identifies altered sensitivity in extremely preterm young adult males and females

**DOI:** 10.1016/j.bja.2018.05.066

**Published:** 2018-07-06

**Authors:** S.M. Walker, H. O'Reilly, J. Beckmann, N. Marlow, the EPICure@19 Study Group

**Affiliations:** 1 Clinical Neurosciences (Pain Research), UCL Great Ormond Street Institute of Child Health, London, UK; 2 Department of Anaesthesia and Pain Medicine, Great Ormond Street Hospital for Children NHS Foundation Trust, London, UK; 3 Academic Neonatology, UCL EGA Institute for Women's Health, London, UK

**Keywords:** conditioned pain modulation, infant, extremely premature, pain

## Abstract

**Background:**

Conditioned pain modulation is a potential biomarker for risk of persistent pain. As early-life experience can alter subsequent somatosensory processing and pain response, we evaluated conditioned pain modulation after extremely preterm birth.

**Methods:**

This observational study recruited extremely preterm (<26 weeks gestation; *n*=98) and term-born control (*n*=48) young adults (19–20 yr) from the longitudinal EPICure cohort. Pressure pain threshold (PPT; variable test stimulus lower leg) was measured before, during, and after a conditioning stimulus (contralateral hand immersion; 5°C water; 30 s). Questionnaires assessed current pain, medication use, anxiety, and pain catastrophising.

**Results:**

For participants tolerating conditioning, there were significant main effects of extremely preterm status, sex, and time on PPT during and after hand immersion. Inhibitory modulation was evoked in 64/98 extremely preterm (3, no change) and 38/48 term-born control (3, facilitation) subjects. The conditioned pain modulation effect (percentage change in PPT) did not differ between the extremely preterm and term-born control groups {53% [95% confidence interval (CI): 41–65] *vs* 57% [95% CI: 42–71]}. Reduced cold tolerance (<20 s) hampered conditioned pain modulation quantification in a higher proportion of extremely preterm participants [extremely preterm *vs* term-born control: 31/98 (32%) *vs* 7/48 (15%); *P*=0.03]. One-third of extremely preterm females withdrew the hand before parallel PPT (<15 s), and had lower baseline PPT than term-born control females [4.9 (95% CI: 4.8–5.1) *vs* 5.3 (95% CI: 5.1–5.5) ln kPa; *P*=0.02]. Higher anxiety, pain catastrophising, and medication use correlated with pain intensity, but not conditioned pain modulation effect.

**Conclusions:**

Cold conditioning evoked inhibitory modulation in the majority of young adults and identified a subgroup of extremely preterm females with increased baseline sensitivity. Early-life experience and sex/gender should be considered when evaluating persistent pain risk with conditioned pain modulation.

**Editor's key points**

• Early-life experience, including preterm birth, may have long-term effects on pain processing.
• Young adults from a longitudinal cohort study of preterm infants were matched to healthy controls.
• Conditioned pain modulation was used to assess descending modulatory effects on pain processing.
• Extremely preterm birth and female sex both affected baseline pain sensitivity and descending modulatory effects.
• The impact of early-life experience and sex on chronic pain vulnerability needs further study.

Conditioned pain modulation (CPM) assesses the ability of a noxious ‘conditioning stimulus’ to alter the sensitivity to a ‘test stimulus’ at a distant body site. Reduced sensitivity or inhibition is the most common response, but a continuum from inhibition to facilitation is possible. Differences in the directionality or degree of CPM have been suggested as a biomarker to predict persistent pain after surgery, risk of chronic pain, or individual differences in treatment response.^1^, ^2^, ^3^, ^4^

The reliability of CPM is influenced by study methodology.^1^ Modulation is quantified by either an alteration in pain threshold (for a variable mechanical, thermal, or electrical test stimulus) or change in pain intensity (to a fixed test stimulus).^1^, ^2^ Cold is a reliable conditioning stimulus,^1^, ^5^ but a range of temperatures (1–13°C) and immersion durations (20–180 s) have been utilized.^5^, ^6^, ^7^, ^8^ The degree of modulation can also be influenced by age,^7^ gender,^9^, ^10^ psychological factors,^2^ differences in baseline sensitivity,^1^ and intercurrent chronic pain.^3^, ^11^

The evaluation of CPM after preterm birth has specific relevance. Early-life pain and tissue injury have been associated with long-term changes in somatosensory processing that can differ with the degree of prematurity, duration of hospitalisation, and pain exposure, and are also influenced by the type and intensity of the subsequent test stimulus.^12^ Anxiety and pain catastrophising scores are higher in preterm than age-matched term-born young adults,^13^, ^14^ and these psychological factors may also influence CPM. Reported associations between preterm birth and chronic pain in later life vary,^12^, ^15^ but CPM may improve the identification of groups with increased risk. One study reported lack of inhibitory modulation in a group of preterm children with higher neonatal pain exposure,^16^ but no studies have assessed CPM in a large cohort of young adults born preterm.

In an observational cohort study, we compared CPM in extremely preterm (EP) and term-born control (TC) young adults. The primary outcome was identification of descending modulatory effects (inhibition, facilitation, and no change) in EP and TC participants. Changes in test stimulus threshold over time identified the directionality and duration of CPM. As the outcome after EP birth is worse in males^17^ and females may have increased chronic pain prevalence and sensitivity to experimental pain stimuli,^18^ sex-dependent differences were assessed. Secondarily, we calculated the percentage change from baseline to quantify the CPM effect. In line with recent recommendations,^12^ correlations between the degree of CPM effect and current pain experience, medication use, and anxiety and pain catastrophising scores were explored.

## Methods

### Study population

The EPICure cohort study recruited all infants born EP before 26 weeks gestational age (GA) across 276 maternity units in the UK and Ireland from March through December 1995. Of 1185 live births, 811 reached the neonatal intensive care unit (NICU), 497 died in the hospital, 314 were discharged home, and 9 have subsequently died.^19^, ^20^ The neurodevelopmental and health outcomes have been longitudinally assessed in EP participants, with recruitment at 30 months,^19^ 6 yr,^21^ 11 yr,^22^ and now at 19 yr^20^ as described previously. Whilst there has been loss to follow-up because of loss of contact details or participant preference, retention over 19–20 yr has been relatively high (92% at 30 months, 68% at 6 yr, 71% at 11 yr, and 42% at 19 yr).^20^ Age-matched TCs were recruited at 6 and 11 yr, and have also provided longitudinal data.^21^ The current study at 19 yr (EPICure@19) was approved by the National Research Ethics Committee Hampshire ‘A’ (Reference: 13/SC/0514). After a written consent, the participants completed general health and cognitive questionnaires, plus respiratory, cardiovascular, and neuroimaging assessments at the University College London Hospitals Clinical Research Facility between February 2014 and October 2015. Current data for demographic variables, cognitive measures, general health, and psychological questionnaires were extracted from the main EPICure database, along with neonatal data for EP participants [weight and GA at birth, clinical risk index for babies (CRIB) score on admission to NICU, and duration of hospital stay]. CPM was performed in conjunction with quantitative sensory testing (QST) on the hand and chest wall, which, along with additional data from the pain history, is reported separately.^23^ Pain and sensory thresholds at 11 yr of age were previously evaluated by the same investigator (S.M.W.) in a subset of the cohort living within 2 h travel of London, but the evaluation at this younger age did not include CPM.^24^

**Fig 1:**
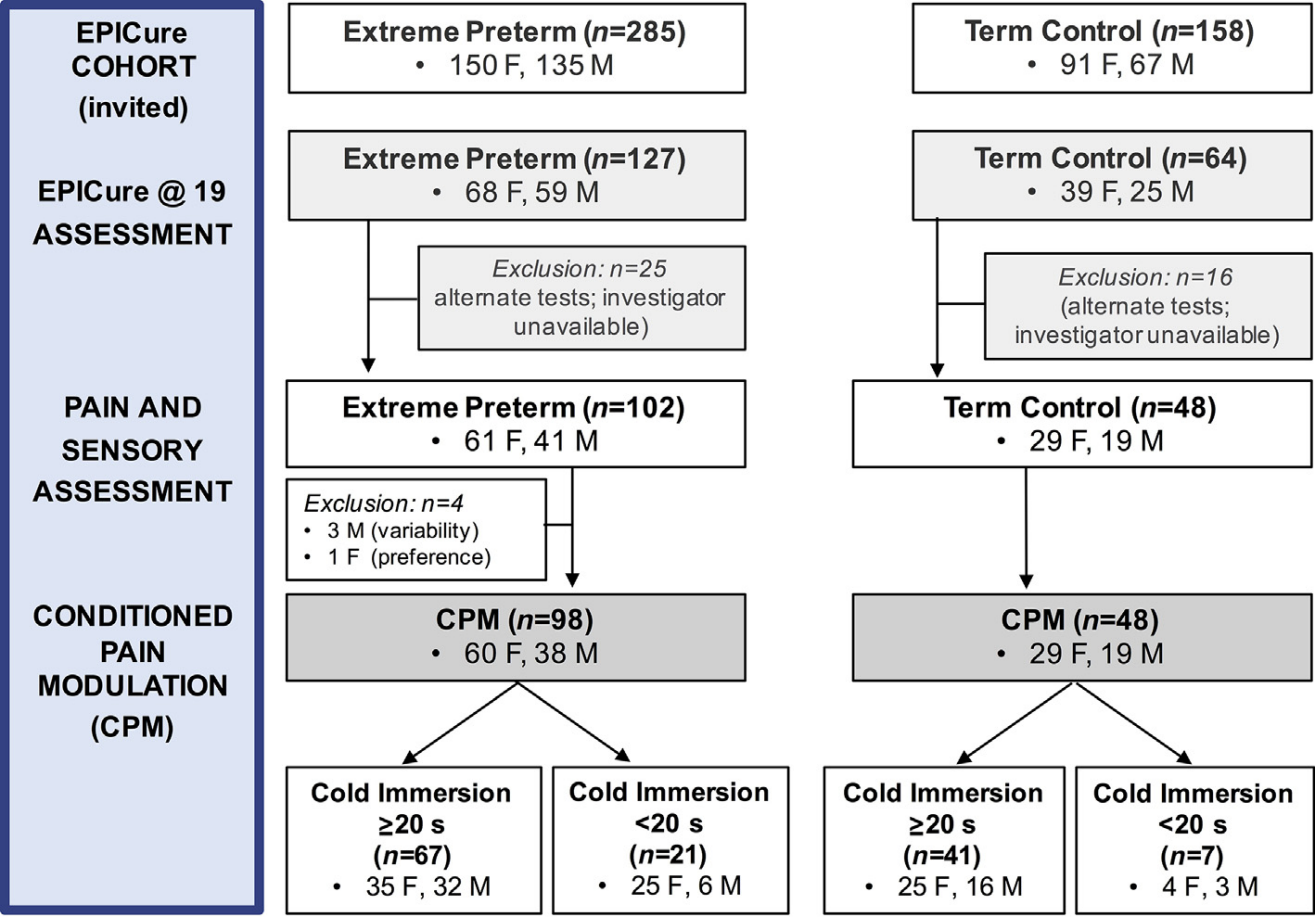
Flow chart of participant numbers and assessments. CPM, conditioned pain modulation; F, female; M, male.

CPM was assessed in 98 EP born and 48 TC participants (Fig. 1) in a dedicated sensory testing facility at UCL Great Ormond Street Institute of Child Health, London. As the assessment included a standardised questionnaire regarding previous and current pain experiences and evaluation of sensory dysfunction related to neonatal scars, the investigator was not blinded to the group. The same standardised verbal instructions were used for testing in the same sequence by a single investigator (S.M.W.).^23^ The participants self-reported VAS measures on linear scales, had control of all response functions (i.e. pressing button for pressure threshold or removing hand from conditioning), and were informed that they could decline or withdraw from testing at any time. All testing was performed at the same time of the day and in the same temperature-controlled room. The participants were offered cool water, but not caffeinated drinks in the 90 min before CPM testing. Throughout this paper, the dichotomous variable of ‘sex’ is reported, as the participants were not asked to self-report their gender. Reporting follows the Strengthening the Reporting of Observational Studies in Epidemiology guidelines.^64^

### Conditioned pain modulation protocol

CPM was assessed using pressure pain threshold (PPT) on the knee as a variable test stimulus and cold water immersion of the contralateral hand as a conditioning stimulus. PPT has good-to-excellent reliability as a variable test stimulus,^1^, ^5^, ^25^, ^26^ and use on the contralateral lower limb ensures engagement of ascending–descending long tract activity and not just segmental spinal inhibitory effects.^1^ PPT testing with cold conditioning is reproducible, sensitive to change,^1^ and has a good test–retest reliability with a smaller sample size than alternative test (electrical and cuff PPT) or conditioning (cuff algometry) stimuli.^27^ As generalised sensitivity may be either increased or decreased after preterm birth depending on participant age, and the type and intensity of test stimulus,^12^, ^28^ PPT was used to provide a reliable linear measure of increases or decreases in baseline threshold. Repeat measures of PPT were performed both in parallel with (15 s), and after cessation of, the conditioning stimulus (50 and 90 s after initial immersion) to assess the duration of effect and minimize the likelihood that the effects are caused by distraction during hand immersion. A cold (5°C) conditioning stimulus with hand immersion for 30 s was chosen, as this evoked inhibitory CPM in healthy 12–17 yr olds,^7^ but is shorter than many protocols, as preterm young adults may have reduced cold-pressor tolerance (5 of 31 withdrew hand before 30 s).^29^

Baseline PPT was the mean of three repetitions of ascending stimuli applied over the head of the right fibula. A computer-controlled handheld 1 cm^2^ algometer (Somedic SENSEBox®, Sosdala, Sweden) incorporating an optical feedback system ensured a standardised increase in pressure (ramp of 40 kPa s^−1^ to a maximum 1000 kPa). The participants pressed a response button when pain/discomfort was perceived. For values at 15 (during conditioning), and 50 and 90 s (after cessation of cold immersion), a single ascending stimulus was applied, with the participants asked to press the button when pain/discomfort was experienced at the same level as baseline.

The left hand was immersed up to the wrist with the palm down and fingers spread into a 5°C circulating water bath (Techne TE-10D Thermoregulator B-8 Bath and RU-200 Dip Cooler; Techne, Burlington, NJ, USA). The participants were instructed to leave their hand in the water for 30 s, or until the stimulus became too uncomfortable/painful.^7^ The duration of immersion was recorded and subjects rated the intensity of hand discomfort (0–10 verbal rating scale) on removal. Survival curves for cold-pressor tolerance are reported,^23^ but, here, data were split based on durations of more, or less than, 20 s.

### Questionnaire-based outcomes

The participants marked VASs (100 mm line) to score average pain in the last week and pretest anxiety (following description of CPM protocol).^7^ Analgesic-use data were extracted from the pain history (S.M.W.),^23^ and additional data, including medications, were extracted from general health (J.B.), and cognitive and psychology questionnaires (H.O.) collected at the University College London Hospital. The Health Utilities Index Mark 3 (HUI-3)^30^ includes self-reported pain ranked as 1=free of pain/discomfort, 2=mild/moderate pain that prevents no activities, 3=moderate pain that prevents a few activities, 4=moderate/severe pain that prevents some activities, and 5=severe pain that prevents most activities. The *Diagnostic and Statistical Manual of Mental Disorders* anxiety *t*-score (range: 50–100; score ≥70 clinically significant) was obtained from the Achenbach Adult Self-Report.^31^ The pain catastrophising scale (PCS)^32^ rates rumination, magnification, and helplessness (total score: 0–52). Wechsler Abbreviated Scale of Intelligence Full Scale IQ (FSIQ) scores were obtained for all participants.^33^

### Statistical analysis

As the EPICure study aimed to recruit the maximum available subjects from this longitudinal cohort and multiple health outcomes were being assessed, no *a priori* power analysis was performed for individual evaluations, such as CPM. In previous CPM studies in healthy young adults, a 5.3% change in PPT with the same algometer was deemed a meaningful CPM effect,^6^ and the current methodology (variable pressure test stimulus and cold conditioning stimulus) had high reliability and the lowest sample size (*n*=17) for detecting significant CPM effects during conditioning (90% power; α=0.5).^27^ The 95% confidence intervals (CIs) for observed changes in PPT are reported in the paper.

Data were analysed using SPSS® version 23 (IBM, Portsmouth, UK), and plotted in Prism version 7 (GraphPad, San Diego, CA, USA); *P*<0.05 was considered statistically significant. Categorical data were compared with Pearson's χ^2^ test or Fisher's exact for smaller samples. After the assessment of normality (D'Agostino and Pearson test), group comparisons were analysed with Student's unpaired two-tailed *t*-test or Mann–Whitney *U*-test. Consistent with previous reports in adolescents and adults,^34^, ^35^ raw PPT data (kPa) were log-transformed. The resultant normally distributed data were analysed for main effects with three-way mixed-design analysis of variance (anova) with two between-subject factors (EP status and sex), and a repeated measures factor of time; degrees of freedom were corrected with Huynh–Feldt estimates of sphericity, and *P* values with Bonferroni adjustment for multiple comparisons.^6^ As sex influences the neurodevelopmental outcome after preterm birth (increased mortality and morbidity in EP males contribute to higher number of females in the sample)^17^ and pain response,^18^ we also evaluated sex differences. To display the time course of modulation and sex-dependent group differences, changes in ln PPT are graphed separately in males and females to identify the change from baseline PPT, and analysed by two-way repeated measures anova with group and time as variables, and multiplicity-adjusted *P* values and Bonferroni *post hoc* comparisons are reported. The percentage change from baseline PPT (kPa) was calculated [(PPT_*x*__seconds_ – PPT_baseline_/PPT_baseline_) × 100] as previously reported,^6^ and provided a normally distributed measure of the degree and direction of change (i.e. ‘CPM effect’). For CPM effect (% change in PPT at 15 s), stepwise linear regression models included candidate variables from Spearman's correlations and prior literature.^8^, ^36^

## Results

CPM was assessed in 98 EP [born between 22.1 and 25.9 (24.9, 0.8; mean, SD) weeks GA, at weight 732,128 g] and 48 TC participants (Table 1). From those attending for somatosensory evaluation, three male EP participants with variable baseline responses (two reported difficulty with numerical scales and prior neonatal surgery, and one was tired and reported difficulty concentrating) had sensory data excluded, and one EP female with Raynaud's symptoms declined cold water immersion.

**Table 1:** Participant characteristics and outcomes based on preterm status and conditioning tolerance. Data are presented as: mean (standard deviation), median [inter-quartile range], or (%). ADHD, attention deficit hyperactivity disorder medication (methylphenidate); antidepr., antidepressant medications (citalopram, fluoxetine, and mirtazapine); Anxiety (Ach), anxiety total score Achenbach Youth Self-Report scale; EP, extremely preterm; F, female; FSIQ, full-scale intelligence quotient Wechsler Abbreviated Scale of Intelligence; Internalising, subscale score Achenbach Youth Self-Report scale; M, male; PCS, pain catastrophising scale total score; PPT, pressure pain threshold; TC, term-born control; VRS, verbal rating scale. ^∗^Group demographic data (EP *vs* TC) are presented for all EP (*n*=102) and TC (*n*=48) in a separate paper^23^ with a subgroup analysis based on sex-dependent differences rather than conditioning stimulus tolerance. ^†^Surgery included: *patent ductus arteriosus* ligation (8F; 4M), inguinal hernia repairs (1F; 7M), laparotomy (3F; 2M), and others (1F; 2M). ^‡^Co-codamol, paracetamol, and codeine. ^¶^Others: migraine prophylaxis (immersion >20 s); azathioprine for Crohn's disease and associated abdominal pain (immersion <20 s). ^§^*P*-values from Student's two-tailed unpaired *t*-test. ^||^*P*-values from two-sided Fisher's exact test. ^#^*P*-values by Mann–Whitney *U*-test. ^∗∗^For measures with missing data, available numbers are listed

	Extremely preterm	Term-born control	*P*	Extremely preterm		*P*	Term-born control		*P*
				Conditioning ≥20 s	Conditioning <20 s		Conditioning ≥20 s	Conditioning <20 s	
	*n*=98	*n*=48		*n*=67	*n*=31		*n*=41	*n*=7	
Characteristics^∗^									
Age (yr) [range]	19.3 (0.6) [18.4–20.5]	19.2 (0.5) [18.1–20.1]	0.3^§^	19.2 (0.6) [18.4–19.3]	19.3 (0.8) [18.5–20.5]	0.5^§^	19.1 (0.5) [18.3–20.1]	19.2 (0.6) [18.1–19.8]	0.6^§^
Height (cm)	163.6 (9.2)	167.3 (8.9)	0.02^§^	165.2 (9.1)	160.1 (8.5)	0.01^§^	167.3 (9.3)	167.3 (7.1)	0.9^§^
Weight (kg)	63.0 (13.9)	67.8 (15.6)	0.06^§^	64.1 (13.2)	60.7 (15.2)	0.3^§^	67.3 (14.5)	70.9 (22.3)	0.6^§^
BMI (kg m^−2^)	23.5 (4.5)	24.1 (4.7)	0.4^§^	23.5 (4.7)	23.5 (4.3)	0.9^§^	23.9 (4.1)	25.3 (7.5)	0.5^§^
Gender; F:M (%F)	61:40 (60%)	29:19 (60%)	0.9^||^	35:32 (52%)	25:6 (81%)	<0.01^||^	25:16 (61%)	4:3 (57%)	0.9^||^
Neonatal surgery	28 (13F; 15M)	0	<0.01^||^	17 (5F; 12M)	11 (8F; 3M)	0.3^||^	0	0	
Sensory data									
Baseline PPT (kPa)	241 [153–376]	213 [160–315]	0.6^#^	336 [290–382]	154 [107–235]	<0.01^#^	206 [158–310]	299 [181–424]	0.3^#^
Baseline PPT, ln	5.5 (0.6)	5.4 (0.5)	0.6^§^	5.6 (0.6)	5.1 (0.5)	<0.01^§^	5.4 (0.5)	5.6 (0.5)	0.3^§^
Immersion time (s)	30 [14–30]	30 [28–30]	0.02^#^	30 [30–30] range=20–30	12 [8–14]	<0.01^#^	30 [30–30] range=21–30	12 [11–13]	<0.01^#^
Immersion pain, VRS 0–10	8 [7.3–10]	8 [7–9]	0.06^#^	8 [7–10]	10 [8–10]	<0.01^#^	8 [7–9]	8 [7–10]	0.7^#^
Questionnaires									
Average pain last week, VAS 0–100 mm	16 (23)	14 (18)	0.5^§^	14 (20)	21 (28)	0.3^§^	14 (19)	16 (13)	0.1^§^
Pretest anxiety, VAS 0–100 mm	7 (16)	1 (3)	0.014^§^	6 (16)	7 (16)	0.8^§^	0.6 (2)	3 (6)	0.6^§^
FSIQ score	88 (14)	104 (10)	<0.01^§^	88 (14)	90 (14)	0.5^§^	105 (10)	98 (7)	0.06^§^
Pain catastrophising (PCS)	5 [0–14.5] (*n*=89)^∗∗^	5 [0–14] (*n*=45)^∗∗^	0.5^#^	8.8 [5.8–11.8] (*n*=61)^∗∗^	6.5 [0–15.5] (*n*=28)^∗∗^	0.6^#^	5 [0–12] (*n*=39)^∗∗^	4.5 [0–28] (*n*=6)^∗∗^	0.8^#^
DSM Anxiety (Ach)	56 [50–58] (*n*=93)^∗∗^	50 [50–54] (*n*=45)^∗∗^	0.011^#^	52 [50–58] (*n*=65)^∗∗^	55 [50–61] (*n*=28)^∗∗^	0.6^#^	50 [50–54] (*n*=39)^∗∗^	50 [50–57] (*n*=6)^∗∗^	0.8^#^
Medication									
Analgesia use	None, 70 (71%); occasional, 20 (20%); regular, 6 (6%) (*n*=97)^∗∗^	None, 37 (77%); occasional, 10 (21%); regular, 1 (2%)	0.4^||^	None, 50 (75%); occasional, 11 (15%); regular, 5 (8%)	None, 19 (%); occasional, 9 (%); regular, 2 (%) (*n*=30)	0.3^||^	None, 30 (73%); occasional, 10 (22%); regular, 1 (2%)	None, 7; occasional, 0 (%); regular, 0 (%)	0.3^||^
Analgesia type	Paracetamol, 19; NSAID, 5; gabapentin and co-codamol,^‡^ 1; others,^¶^ 2	Paracetamol, 6; NSAID, 4; NSAID and co-codamol, 1		Paracetamol, 12; NSAID, 3; gabapentin and co-codamol, 1; others, 1	Paracetamol, 7; NSAID, 2; gabapentin and co-codamol, 1; others, 1		Paracetamol, 6; NSAID, 4; NSAID + co-codamol, 1	None	
Psychotropic medication	9 (10%) (7F, 2M) (*n*=92)^∗∗^	1 (2%) (1M) (*n*=44)^∗∗^	0.17^||^	Antidepr., 5 (4F; 1M); ADHD, 1F	Antidepr., 3 (2F; 1M)		Antidepr., 1 (M)	None	

### Baseline sensitivity

Baseline PPT over the fibula head did not differ significantly between the EP and TC groups (Table 1). However, within the EP group, baseline PPT was lower in females than males [5.2 ln kPa (95% CI: 5.1–5.4) *vs* 5.8 (95% CI: 5.7–6.0)], with the greatest difference in EP males with prior neonatal surgery [6.0 ln kPa (95% CI: 5.7–6.2); *n*=15], as also reported for PPT on the digit in these participants.^23^ Tolerance of cold conditioning was significantly shorter in EP participants, and a higher proportion of EP [EP *vs* TC: 31/98 (32%) *vs* 7/48 (85%)] participants withdrew the hand before 20 s (*P*=0.03) (Table 1). Shorter duration of immersion correlated with a higher pain score on hand removal and lower baseline PPT (Table 2), predominantly in EP participants (Supplementary Table S2).

**Table 2:** Correlations between sensory variables, current pain, psychological variables, and medication use (all participants; *n*=146). CPM, conditioned pain modulation; DSM-Anxiety, anxiety *t*-score Achenbach Youth Self-Report scale; HUI-3, Health Utilities Index Mark 3; PCS, pain catastrophising scale total score; PPT, pressure pain threshold; VRS, verbal rating scale. Data = two-tailed Spearman's rho bivariate correlation coefficient *P<0.05 **P<0.01

	Baseline PPT	Immersion time	Immersion pain	CPM %	Pain ranking	Regular analgesia	PCS	Anxiety	Regular psychotropics
Baseline PPT (ln kPa)	1.0								
Immersion time (s)	0.30^∗∗^	1.0							
Immersion pain (VRS)	–0.27^∗∗^	–0.36^∗∗^	1.0						
Conditioned pain modulation % (15 s)	–0.30^∗∗^	–0.20^∗∗^	–0.06	1.0					
Pain ranking (HUI-3) [*n*=139]	–0.12	–0.08	0.05	–0.01	1.0				
Regular analgesia	–0.11	–0.09	0.07	0.03	0.31^∗∗^	1.0			
Pain catastrophising [*n*=134]	–0.15	–0.04	0.12	–0.16	0.23^∗∗^	0.26^∗∗^	1.0		
DSM-Anxiety [*n*=138]	–0.08	–0.05	0.11	–0.02	0.27^∗∗^	0.20^∗^	0.40^∗∗^	1.0	
Regular psychotropics [*n*=135]	–0.06	–0.01	0.16	0.10	0.19^∗^	0.20^∗^	0.14	0.33^∗∗^	1.0

### Detection of modulatory effect

**Fig 2:**
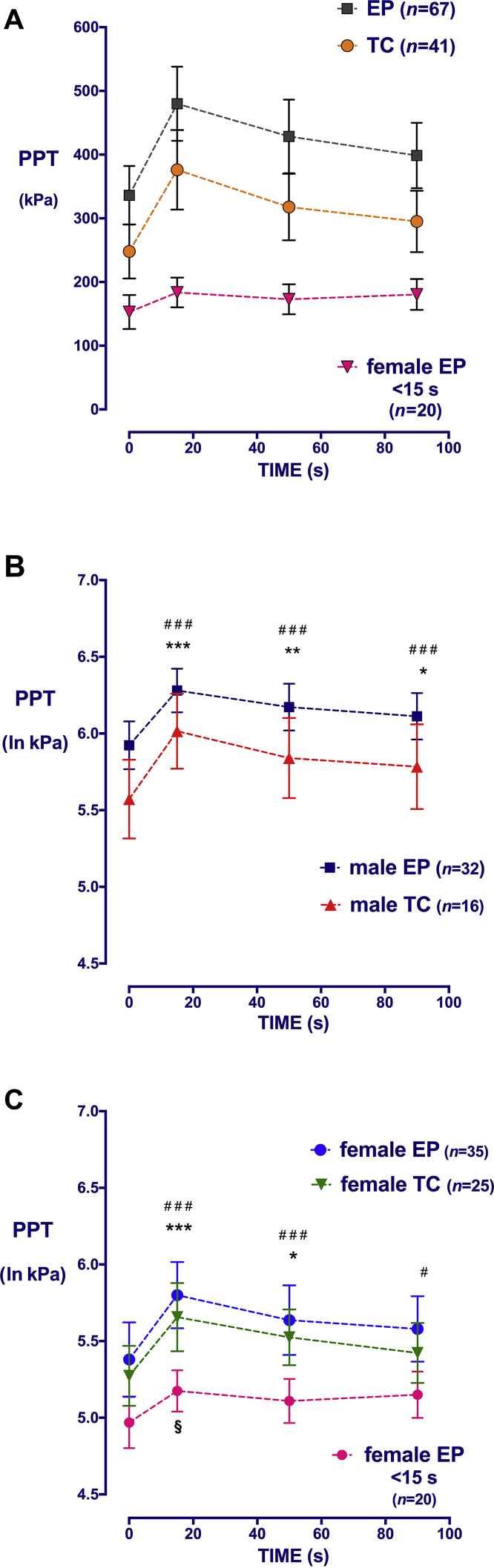
Effect of preterm birth, sex, and time on pressure pain threshold during conditioned pain modulation (CPM). (a) Change in mechanical pressure pain threshold over the right fibula head (PPT, raw data, kPa) in term control (TC) and extremely preterm (EP)-born young adults during (15 s) and after (50 and 90 s) a conditioning stimulus (0–30 s). Females unable to tolerate immersion until the parallel PPT measurement (<15 s) had no significant change in PPT with time. Additional groups with <15 s immersion were too small for analysis (five EP males, four TC females, and two TC males). Data points=mean [95% confidence interval (CI)]. (b) and (c) Change in log-normalised PPT (ln kPa) with time in (b) males and (c) females. For EP and TC participants tolerating at least 20 s conditioning, PPT is significantly increased above baseline at 15 and 50 s. In EP females with <15 s immersion, a minor increase in threshold is seen only at 15 s. Data points=mean (95% CI). ^###^*P*<0.001, ^#^*P*<0.05, ***P<0.001, **P<0.01, *P<0.05, §*P*<0.05; two-way repeated measures analysis of variance with Bonferroni *post hoc* comparisons of within-group change compared with baseline.

Twenty seconds was chosen as a cut-off for adequate conditioning. This is the minimum duration previously reported in CPM protocols, and ensured hand removal occurred after, rather than during or before, the parallel 15 s PPT measurement. The duration of immersion between 20 and 30 s reliably evoked modulation (at least 10% change in PPT) in all TC participants (38, inhibition; 3, facilitation) and in 64 EP participants (64, inhibition; 3, no change). After 20 s of conditioning, group data confirm significant increases at 15 s in both TC [5.4 (95% CI: 5.2–5.5) to 5.8 (95% CI: 5.6–6.0) ln kPa] and EP [5.6 (95% CI: 5.5–5.8) to 6.0 (5.9–6.2)] groups, which were maintained beyond cessation of the stimulus (Fig. 2a). There was a significant main effect of EP status (*F*_1,104_=4.8; *P*=0.03), time (*F*_2.8,265_=76.7; *P*<0.001), and sex (*F*_1,104_=17.7; *P*<0.001) on PPT ln kPa (Fig. 2b and c).

### Increased sensitivity in a subgroup of extremely premature females

To identify the factors associated with reduced conditioning tolerance, we compared measures in participants who did, or did not, tolerate 20 s cold immersion (Table 1). EP young adults with reduced conditioning tolerance (<20 s) also had higher pain scores on hand removal and lower baseline PPT, and a higher proportion were females (Table 1). Twenty-eight EP (and no TC) participants had required neonatal surgery and were distributed across both immersion durations (see Table 1 for sex distribution and type of surgery).

As PPT may not be accurate if the participants are removing and reporting VAS in one hand, whilst they are pressing a PPT response button with the contralateral hand, data related to immersion times of 15–19 s (five EP females, one EP male, and one TC male) were not further analysed. The remaining 31 participants (20 EP females, 5 EP males, 4 TC females, and 2 TC males) tolerated less than 15 s immersion and removed the hand before the first PPT. Only EP females comprised a sufficient sample for further analysis. Brief immersion failed to produce modulatory effects in this group, as there was no change in raw PPT with time (Fig. 2a), and only a minor increase in normalised PPT (*P*<0.05) at 15 s that was not maintained at 50 s (Fig. 2c).

### Degree of conditioned pain modulation

**Fig 3:**
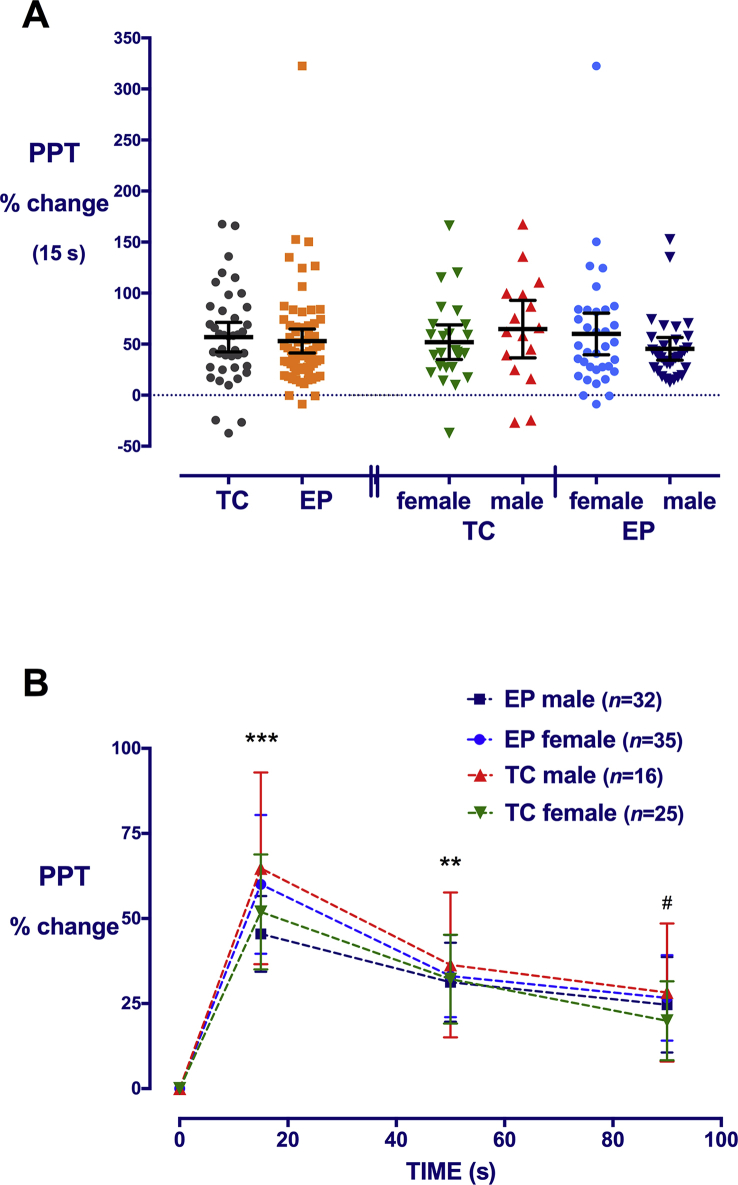
Degree of conditioned-pain-modulation effect after at least 20 s conditioning stimulus in extremely preterm (EP) and term-born control (TC) males and females. (a) The percentage change in pressure pain threshold (PPT) during the conditioning stimulus is not significantly different across groups based on EP status or sex. Individual data points, bars=mean [95% confidence interval (CI)]. (b) Raised PPT during the conditioning stimulus (15 s) is maintained at 50 and 90 s. Data points=mean (95% CI); ***P<0.001; **P<0.01 all groups increase *vs* baseline; #*P*<0.05 TC males, EP females, and EP males *vs* baseline; two-way repeated measures analysis of variance with Bonferroni *post hoc* comparisons.

To compare the degree of CPM effect, the percentage change from baseline was calculated for individual participants tolerating at least 20 s immersion. The degree of CPM effect varied with time, with a similar maximal change at 15 s in both EP and TC groups [53% (95% CI: 41–65) *vs* 57% (95% CI: 42–71)] (Fig. 3). Whilst PPT was higher in EP males than EP females at all time points and the absolute change during conditioning was slightly greater [133 kPa (inter-quartile range: 94–225) *vs* 89 (51–196); *P*=0.048], once expressed as percentage change from the higher baseline, there were no sex differences in CPM effect. Baseline PPT (ln kPa) was negatively correlated with CPM effect (% change at 15 s) (Table 2), and when separated by group, in EP [*r*=–0.45 (95% CI: –0.6 to –0.18; *P*<0.01] (Supplementary Table S1B), but not TC (*r*=–0.18; *P*=0.3) participants. In regression analysis with participants tolerating 20–30 s immersion (*n*=108), the duration of conditioning did not influence CPM effect (therefore, these durations were grouped together), but PPT had a significant effect (Table 3). Reduced cold tolerance is most marked in EP females, particularly those with prior neonatal surgery,^23^ and shorter immersion time correlates with lower PPT in EP, but not TC participants (Supplementary Table S2). Including all participants in the regression model (*n*=148) highlighted the impact of shorter immersion time, as this variable now had a significant impact on calculated CPM effect, but there were no marked changes related to other variables (Supplementary Table S3).

**Table 3:** Linear model of conditioned-pain-modulation effect (% change in PPT at 15 s) for participants with conditioning tolerance ≥20 s (*n*=108). DSM-Anxiety, anxiety *t*-score Achenbach Youth Self-Report scale; HUI-3, Health Utilities Index Mark 3; PCS, pain catastrophising scale; PPT, pressure pain threshold; SE, standard error

Variables	Step 1 (*n*=108)				Step 2 (*n*=108)				Step 3 (*n*=96)			
	*B*	SE *B*	β	*P*	*B*	SE *B*	β	*P*	*B*	SE *B*	β	*P*
Baseline PPT	–27	7.1	–0.35	<0.001	–30	7.9	–0.39	<0.001	–35	8.6	–0.43	<0.001
Immersion time	–1.9	1.5	–0.12	0.19	–1.7	1.5	–0.10	0.27	–0.16	1.6	–0.01	0.92
Extremely premature status					–1.8	9.0	–0.02	0.84	2.6	9.7	0.02	0.79
Sex					–8.9	9.3	–0.09	0.34	–14	10	–0.14	0.19
Pain (HUI-3 ranking)									–5.4	7.5	0.08	0.47
Regular analgesics									21	24	0.09	0.38
Catastrophising (PCS)									–0.58	0.56	–0.13	0.30
DSM-Anxiety (Ach)									–0.57	0.59	–0.12	0.33
Regular psychotropics									65	23	0.29	0.01
*R*^2^	0.15				0.16				0.29			
*F* for *R*^2^	*F*_2,105_=9.3; *P*=0.001				*F*_4,103_=4.8; *P*=0.001				*F*_9,87_=3.9; *P*=0.001			

### Current pain, medication, and psychological outcomes are related, but do not influence CPM effect

Higher self-reported pain (average VAS in last week or HUI-3 ranking), regular analgesia use, higher anxiety and catastrophising scores, and regular psychotropic medications were inter-related, but did not correlate with CPM variables (baseline PPT, immersion duration, immersion pain, or CPM effect) (Table 2).

Medication use is listed in Table 1. No participants had taken analgesia on the day of testing, and 27/97 EP and 11/48 TC reported use of occasional or regular analgesia, most often paracetamol or an NSAID (Table 1). Pain-related conditions had required specialist management in four EP females (steroid injection for knee pain, two pain clinic reviews and gabapentin for persistent post-surgical pain or fibromyalgia, and rheumatology review and physiotherapy for back pain), two EP males (neurologist and migraine prophylaxis, and gastroenterologist and azathioprine for Crohn's disease and associated abdominal pain), and one TC female (rheumatologist and physiotherapy for hypermobility).

Self-reported medication use (general health questionnaire) was available for 136 participants. Ten participants (nine EP and one TC) reported medications with psychoactive properties (antidepressants or medications used for attention deficit hyperactivity disorder; Table 1). Participants taking antidepressant medications had higher anxiety scores (*P*<0.01) (Supplementary Table S1). Seven of these participants tolerated at least 20 s immersion and tended to have a higher CPM effect, but the variability is wide (one, no change; six, inhibitory response; 99±106% at 15 s). Inclusion of psychotropic medications had a significant effect in the regression model (Table 3; Supplementary Table S3), but numbers are small, and significance was lost when an outlier with a very low baseline PPT (48 kPa, EP female) and high percentage change during conditioning (PPT 200 kPa; 332% increase; see Fig. 3a) was excluded.

Although FSIQ was lower in EP participants (Table 1), FSIQ did not correlate with conditioning tolerance, baseline PPT, or CPM effect (Supplementary Table S2). Height and weight were lower in EP than TC participants, but BMI did not differ (Table 1) and did not influence CPM parameters. Prior neonatal surgery influenced baseline PPT in EP males and conditioning tolerance in females,^23^ but not CPM effect. Neonatal variables (birth weight, CRIB score on admission to intensive care, and duration of hospital stay) also did not correlate with CPM parameters (Supplementary Table S2A).

## Discussion

Inhibitory CPM was demonstrated in the majority of young adults, but differences in conditioning stimulus tolerance influenced the ability to quantify CPM. In participants tolerating conditioning, there were significant main effects of EP status, sex, and time on PPT during and after hand immersion, with inhibitory modulation evoked in 64/98 EP (3, no change) and 38/48 TC (3, facilitation). Identification and quantification of CPM in EP, but not TC, participants were influenced by sex-dependent differences in sensitivity to both the test (reduced sensitivity in EP males) and conditioning stimulus (increased sensitivity in EP females). One-third of EP females had low baseline PPT and reduced cold-pressor tolerance, and the brief conditioning did not alter the subsequent PPT. Current pain, anxiety, and pain catastrophising scores did not correlate with CPM magnitude.

Baseline sensitivity and conditioning tolerance were significantly influenced by EP status and sex. Previous comparison to TCs reported lower PPT in very preterm (VP; mean: 31 weeks GA) adolescents, predominantly as a result of increased sensitivity in females and minimal difference in males.^28^ Here, PPT on the head of the fibula was lower in EP females than EP males, and correlated with sex-dependent differences measured on the middle digit of the hand in this cohort.^23^ Reduced cold-pressor tolerance has also been reported in young adults born EP (mean: 26.8 weeks GA) with 5 of 31 withdrawing the hand before 30 s, and females were more sensitive.^29^ In VP (mean: 31 weeks GA) young adults, female sex and neonatal necrotising enterocolitis reduced the likelihood of tolerating cold immersion at 19 yr,^37^ but details of surgery for this or other conditions are not reported. We similarly found reduced sensitivity in EP females, particularly those that had undergone neonatal surgery,^23^ but the proportion tolerating less than 20 s immersion was larger than anticipated, and brief immersion hampered our ability to reliably quantify CPM effect.

CPM can clearly identify differences in the proportion of participants with inhibition, no change, or facilitation.^38^, ^39^ For participants tolerating immersion, significant inhibitory CPM was identified in the majority of both EP and TC participants, and the smaller numbers with no change or facilitation did not differ between groups. Whilst these descriptive differences are generalisable across studies, different methods and time points have been used to compare the magnitude of CPM across groups.^4^, ^16^, ^36^, ^40^, ^41^ Despite clear group increases in PPT after conditioning, there was a wide within-group variability in calculated percent CPM, as also seen in some previous evaluations in healthy adolescents^36^ and adults.^42^ This limited our ability to identify group differences in CPM magnitude. Whilst there are no generally accepted ‘normative’ data for the magnitude of CPM effect, our data (mean increase in PPT of 50–65% at 15 s, and 31–36% at 50 s in TC) are consistent with studies using similar methodology in adults^9^, ^43^ and adolescents,^7^, ^36^ and persistence beyond the conditioning stimulus suggests CPM is not solely related to non-specific distraction.^3^

Preterm birth has been associated with persistent alterations in somatosensory function and pain response,^12^, ^23^ but CPM has only previously been assessed in a small group of VP children. CPM efficacy varies with age,^1^ and weak inhibitory effects in childhood become more robust throughout adolescence.^7^ Mechanisms underlying this delayed maturation of descending inhibition have been identified in rodents,^12^ and this normal developmental trajectory can be altered by neonatal tissue injury (hind-paw carrageenan inflammation^44^ or incision^45^). At 7–11 yr, inhibitory CPM was enhanced (greater decrease in heat pain intensity after cold conditioning) in a ‘low-pain’ group of six children born VP (28–32 weeks gestation), but absent in a ‘high-pain’ group of seven with longer NICU admission and increased procedural pain exposure.^16^ The EPICure cohort participants were born at an earlier GA, had longer hospital stay, and 28% required neonatal surgery, suggesting they would constitute a high-pain group.^16^ However, in these EP young adults, failure to tolerate the conditioning stimulus and female sex, rather than neonatal variables *per se*, were the predominant factors associated with ‘absence’ of CPM.

Increased inhibitory CPM has been reported in males.^9^, ^18^, ^42^ Whilst the relative change in PPT was higher in EP males, once expressed as percentage change from the higher PPT, this difference was lost. Females tend to have lower PPT^6^, ^46^ and reduced cold-pressor tolerance,^47^, ^48^ and CPM identified a large subset of EP females with increased sensitivity to both pressure and cold immersion. This ‘lack’ of CPM response is likely to reflect failure of brief immersion to engage descending modulatory effects.^49^ There is currently no standardised reporting for ‘non-responders’ or subjects with no change in CPM,^1^, ^50^ and data from subjects with reduced conditioning tolerance are often excluded.^6^, ^36^ As this included a large number and proportion of our EP females, data from this group are presented separately. The degree to which the duration and intensity of the conditioning stimulus influence the CPM effect is debated.^42^ A CPM paradigm that alters the conditioning intensity based on individual sensitivity^40^ would have advantages for groups, such as EP young adults, who have marked variability in conditioning tolerance.

Psychological factors influence descending modulatory pathways and interact with similar neurotransmitter systems as CPM.^2^, ^39^ A meta-analysis found no overall correlation between CPM and psychological variables in healthy or pain populations, but a secondary analysis showed modality-specific correlations between increased CPM effect and higher anxiety using pressure-based testing and higher pain catastrophising using an electrical test stimulus.^2^ Children born VP had higher pain catastrophising scores,^14^ and altered patterns of functional MRI activation by a prolonged thermal heat stimulus at 11–16 yr included differences in brainstem modulatory regions.^51^ Consistent with existing literature,^13^, ^52^ our EP young adults self-reported more internalising and anxiety, and pain catastrophising was higher in females, but whilst these measures were associated with increased self-reported pain, they did not correlate with cold tolerance or CPM effect. A large Norwegian population-based registry found adults born VP or EP were more likely to be taking psychotropic medications (antidepressants, anxiolytics, and hypnotics), with overall greater use by females, and EP males more likely to be taking medication for attention deficit hyperactivity disorder.^53^ Here, the EP participants taking psychotropic medications had higher self-reported anxiety and pain, and inclusion of this variable influenced the regression model, but the small numbers and wide variability limited the reliability of relationships with the CPM effect.

Reduced inhibitory CPM^3^, ^11^ or a shift to facilitation has been reported in adults^38^, ^40^ and youth^41^ with chronic pain. Here, there were no clear associations between current pain experience and the degree or directionality of CPM. Whilst restoration of inhibition after treatment suggests that impaired CPM is a reversible effect of chronic pain,^1^ reduced inhibitory CPM in adults^54^ and facilitation rather than inhibition in adolescents^8^ predicted persistent musculoskeletal pain. Similarly, impaired preoperative CPM has predicted persistent post-surgical pain after different types of surgery.^4^, ^55^, ^56^, ^57^ EP females with increased sensitivity to pressure and noxious cold, in whom robust inhibition could not be quantified, may be at increased risk of persistent pain after surgery or injury in the future. An ongoing assessment in large cohorts is required to further quantify the risk and evaluate the potential preventive interventions.

The limitations of this study include potential selection bias, as not all EPICure cohort participants were assessed at 19–20 yr. EPICure@19 participants did not differ in birth weight, GA, or sex from those lost to follow-up, and those attending for QST did not differ from the remaining participants undergoing other assessments at 19 yr.^23^ Long-term follow-up tends to favour NICU survivors with a relatively favourable outcome,^58^ and EPICure@19 participants had higher socio-economic status and higher mean IQ scores at earlier assessments than non-participants,^20^ suggesting that the effects may be underestimated. Ethnicity was not assessed, as the majority of subjects were Caucasian, and fewer EP males were tested, but with a matched proportion of controls. In females, the results were not stratified by menstrual phase or use of oral/implanted contraceptive hormones, although some reports suggest these factors have minimal effect on CPM magnitude,^9^ pressure threshold, or cold-pressor sensitivity.^59^ The analysis of CPM effect tends to focus, as here, on bulbospinal control of descending inhibition/facilitation; however, additional mechanisms that may also modulate pain response and be influenced by preterm birth include alterations in hypothalamic–pituitary–adrenal axis function^60^ and autonomic nervous system activation.^38^, ^61^

In summary, descending inhibitory modulation was identified in the majority of participants, with increases in PPT during conditioning maintained beyond the stimulus. For those tolerating cold immersion, the degree and directionality of CPM did not differ between EP and term-born young adults. However, the ability to quantify and compare the CPM effect was influenced by sensitivity to the test and the conditioning stimulus. Improvements in neonatal intensive care are now resulting in increased numbers of EP born children reaching adulthood, and identifying risk factors for future illness is a major focus of longitudinal outcome studies.^62^, ^63^ The CPM protocol identified a high proportion of EP females with a persistent increased sensitivity to pressure and noxious cold that may influence future pain experience or risk of persistent pain.^23^ As sex/gender and preterm birth influence conditioning and test stimulus sensitivity, these factors should be considered when choosing the methodology and analysis of CPM to predict or assess persistent post-surgical pain or chronic pain.

## Authors' contributions

Study design/planning: S.M.W., H.O., J.B., N.M.

Data analysis: S.M.W., H.O., J.B.

Drafting and writing paper: S.M.W.

Revision and approval of the final manuscript: S.M.W., H.O., J.B., N.M.

Overall planning and conduct of evaluations at 19: EPICure@19 study group.
