# Supplementary material for: Conditioned pain modulation identifies altered sensitivity in extremely preterm young adult males and females
Source: Br J Anaesth. 2018 Jul 6;121(3):636–46. doi: 10.1016/j.bja.2018.05.066 (PMC6200113; doi:10.1016/j.bja.2018.05.066)
Supplement: Multimedia component 1 [file mmc1.docx]

**Supplementary Table 1** Correlations with degree of Conditioned Pain Modulation in subgroups based on immersion duration (A) or all extreme preterm birth participants (B).

A: EP and TC subjects tolerating immersion >/=20 secs

|  | **Baseline PPT** | **Immersion time** | **Immersion pain** | **CPM %** | **Pain ranking** | **Regular analgesia** | **PCS** | **Anxiety** | **Regular psychotropics** |
| --- | --- | --- | --- | --- | --- | --- | --- | --- | --- |
| **Baseline PPT (*ln* kPa)** | 1.0 |  |  |  |  |  |  |  |  |
| **Immersion time (s)** | 0.09 | 1.0 |  |  |  |  |  |  |  |
| **Immersion pain (VRS)** | -0.25** | -0.24* | 1.0 |  |  |  |  |  |  |
| **CPM % (15 sec)** | -0.32** | 0.04 | -0.06 | 1.0 |  |  |  |  |  |
| **Pain ranking (HUI-3)** [*n*=104] | -0.09 | -0.08 | 0.02 | 0.01 | 1.0 |  |  |  |  |
| **Regular analgesia** | -0.12 | -0.12 | 0.03 | 0.08 | 0.35** | 1.0 |  |  |  |
| **Pain Catastrophizing** [*n*=100] | -0.15 | 0.01 | 0.06 | 0.01 | 0.30** | 0.24* | 1.0 |  |  |
| **DSM-Anxiety** [*n*=104] | -0.06 | 0.01 | 0.08 | 0.21* | 0.20* | 0.40** | 0.40** | 1.0 |  |
| **Regular psychotropics** [*n*=101] | -0.05 | 0.03 | 0.17 | 0.14 | 0.25* | 0.33** | 0.03 | 0.28** | 1.0 |

B: All EP only

| **EP (n=98)** | **Baseline PPT** | **Immersion time** | **Immersion pain** | **CPM %** | **Pain ranking** | **Regular analgesia** | **PCS** | **Anxiety** | **Regular psychotropics** |
| --- | --- | --- | --- | --- | --- | --- | --- | --- | --- |
| **Baseline PPT (*ln* kPa)** | 1.0 |  |  |  |  |  |  |  |  |
| **Immersion time (s)** | 0.45** | 1.0 |  |  |  |  |  |  |  |
| **Immersion pain (VRS)** | -0.38** | -0.38* | 1.0 |  |  |  |  |  |  |
| **CPM % (15 sec)** | -0.31** | 0.16 | -0.03 | 1.0 |  |  |  |  |  |
| **Pain ranking (HUI-3)** [*n*=94] | -0.17 | -0.08 | 0.06 | 0.05 | 1.0 |  |  |  |  |
| **Regular analgesia** | -0.16 | -0.11 | 0.11 | -0.01 | 0.29** | 1.0 |  |  |  |
| **Pain Catastrophizing** [*n*=89] | -0.24* | -0.09 | 0.16 | -0.05 | 0.29** | 0.29** | 1.0 |  |  |
| **DSM-Anxiety** [*n*=93] | -0.10 | 0.01 | 0.01 | 0.03 | 0.31** | 0.16 | 0.43** | 1.0 |  |
| **Regular psychotropics** [*n*=91] | -0.09 | 0.01 | 0.21 | 0.13 | 0.24* | 0.21 | 0.22* | 0.38** | 1.0 |

Data = two-tailed Spearman’s rho bivariate correlation co-efficient: *correlation significant at 0.05; **correlation significant at 0.01 level

*Legend*: CPM %, conditioned pain modulation % change from baseline at 15 seconds; PCS, Pain Catastrophizing Scale total score DSM-Anxiety, anxiety total score Achenbach Youth Self-Report scale

**Supplementary Table 2.** Correlations between degree of Conditioned Pain Modulation, immersion time, current pain and psychological variables based on EP status

**(A)** Extremely Preterm born young adults (all participants)

| **EXTREMELY PRETERM [n=98]** | **CPM %** | **Baseline PPT** | **Immersion time** | **Immersion pain** | **Pain experience** | **Pain VAS** | **Pre-test anxiety** | **Anxiety**  **(T-Ach)** | **PCS** | **FSIQ** | **BMI** | **Time hosp** | **CRIB score** | **Birth weight** |
| --- | --- | --- | --- | --- | --- | --- | --- | --- | --- | --- | --- | --- | --- | --- |
| **CPM % (15 sec)** | 1.0 |  |  |  |  |  |  |  |  |  |  |  |  |  |
| **Baseline PPT (kPa)** | -0.31** | 1.0 |  |  |  |  |  |  |  |  |  |  |  |  |
| **Immersion time (s)** | 0.16 | 0.45** | 1.0 |  |  |  |  |  |  |  |  |  |  |  |
| **Immersion pain (VRS)** | -0.03 | -0.38** | -0.38** | 1.0 |  |  |  |  |  |  |  |  |  |  |
| **Pain ranking (HUI-3)** [*n*=94] | 0.05 | -0.17 | -0.07 | 0.06 | 1.0 |  |  |  |  |  |  |  |  |  |
| **Ave Pain (VAS 0-100)** | -0.03 | -0.18 | -0.11 | 0.06 | 0.48** | 1.0 |  |  |  |  |  |  |  |  |
| **Pre-test anxiety (0-100)** | 0.01 | -0.12 | -0.02 | 0.01 | 0.03 | 0.20 | 1.0 |  |  |  |  |  |  |  |
| **DSM-Anxiety** [*n*=93] | 0.03 | -0.10 | 0.01 | 0.01 | 0.31** | 0.19 | 0.30** | 1.0 |  |  |  |  |  |  |
| **Pain Catastrophizing** [*n*=89] | -0.05 | -0.24* | -0.09 | 0.16 | 0.29** | 0.24* | 0.23* | 0.43** | 1.0 |  |  |  |  |  |
| **FSIQ** | 0.09 | -0.14 | -0.05 | -0.05 | -0.33** | -0.25* | -0.19 | -0.15 | -0.05 | 1.0 |  |  |  |  |
| **BMI** | -0.11 | 0.15 | -0.04 | -0.05 | 0.03 | 0.12 | -0.12 | 0.01 | 0.10 | -0.08 | 1.0 |  |  |  |
| **Time hospital** [*n*=78] | -0.18 | -0.13 | -0.08 | 0.04 | 0.01 | 0.23 | 0.06 | 0.03 | 0.10 | -0.29** | -0.04 | 1.0 |  |  |
| **CRIB score** [*n*=95] | -0.17 | -0.12 | -0.09 | 0.07 | 0.11 | 0.25* | 0.16 | -0.12 | -0.05 | -0.18 | -0.02 | 0.38** | 1.0 |  |
| **Birth weight** [*n*=98] | 0.03 | 0.15 | -0.03 | -0.07 | -0.05 | -0.19 | -0.19 | 0.05 | -0.04 | 0.15 | -0.09 | -0.32** | -0.62** | 1.0 |

**(B)** Term-born control young adults (all participants)

| **TERM CONTROL [n=48]** | **CPM %** | **Baseline PPT** | **Immersion time** | **Immersion pain** | **Pain ranking** | **Pain VAS** | **Pre-test anxiety** | **Anxiety**  **(Ach)** | **PCS** | **FSIQ** | **BMI** |
| --- | --- | --- | --- | --- | --- | --- | --- | --- | --- | --- | --- |
| **CPM % (15 sec)** | 1.0 |  |  |  |  |  |  |  |  |  |  |
| **Baseline PPT (kPa)** | -0.28 | 1.0 |  |  |  |  |  |  |  |  |  |
| **Immersion time (sec)** | 0.26 | -0.09 | 1.0 |  |  |  |  |  |  |  |  |
| **Immersion pain (VRS 0-10)** | -0.12 | -0.06 | -0.24 | 1.0 |  |  |  |  |  |  |  |
| **Pain ranking (HUI-3)** [*n*=45] | -0.08 | 0.04 | 0.04 | -0.03 | 1.0 |  |  |  |  |  |  |
| **Ave. Pain (VAS 0-100)** | -0.11 | 0.12 | -0.13 | -0.03 | 0.18 | 1.0 |  |  |  |  |  |
| **Pre-test anxiety (0-100)** | -0.21 | 0.15 | -0.08 | 0.05 | -0.23 | 0.12 | 1.0 |  |  |  |  |
| **DSM-Anxiety** [*n*=45] | 0.01 | -0.06 | -0.03 | 0.33* | 0.04 | 0.13 | -0.04 | 1.0 |  |  |  |
| **Pain Catastrophizing** [*n*=45] | 0.05 | 0.04 | 0.14 | 0.01 | 0.07 | 0.23 | 0.20 | 0.28 | 1.0 |  |  |
| **FSIQ** | 0.09 | -0.14 | 0.21 | -0.37* | -0.18 | -0.03 | 0.20 | -0.24 | -0.08 | 1.0 |  |
| **BMI** | -0.15 | 0.10 | -0.04 | 0.19 | 0.17 | 0.12 | -0.24 | 0.23 | -0.32 | -0.21 | 1.0 |

Data = two-tailed Spearman’s rho bivariate correlation co-efficient: *correlation significant at 0.05; **correlation significant at 0.01 level

*Legend*: CPM %, conditioned pain modulation % change from baseline at 15 seconds; DSM-Anxiety, anxiety total score Achenbach Youth Self-Report scale; Internalizing (Ach), internalizing subscale score Achenbach Youth Self-Report scale; PCS, Pain Catastrophizing Scale total score

**Supplementary Table 3.** Linear model of CPM Effect (% change in PPT at 15 secs; all participants irrespective of conditioning tolerance)

|  | **Step 1** (n=145) | | | | **Step 2** (n=145) | | | | **Step 3** (n=127) | | | |
| --- | --- | --- | --- | --- | --- | --- | --- | --- | --- | --- | --- | --- |
| **Variables** | **B** | **SE B** | **β** | ***p*** | **B** | **SE B** | **β** | ***p*** | **B** | **SE B** | **β** | ***p*** |
| Baseline PPT (*ln* kPa) | -31 | 6.1 | -.40 | <0.001 | -35 | 6.7 | -.46 | <0.001 | -37 | 7.3 | -.47 | <0.001 |
| Immersion time (s) | 1.3 | 0.45 | .24 | 0.003 | 1.4 | 0.47 | .24 | 0.004 | 1.4 | 0.5 | .23 | 0.008 |
| EP status |  |  |  |  | -0.17 | 7.7 | -.002 | 0.98 | 4.2 | 8.4 | .04 | 0.61 |
| Sex |  |  |  |  | -12.5 | 8.0 | -.13 | 0.12 | -14.8 | 8.9 | -.15 | 0.09 |
| Pain (HUI-3 ranking) |  |  |  |  |  |  |  |  | 4.4 | 5.8 | .07 | 0.46 |
| Regular analgesics |  |  |  |  |  |  |  |  | 21 | 19 | .10 | 0.29 |
| Catastrophizing (PCS) |  |  |  |  |  |  |  |  | -0.53 | 0.46 | -.12 | 0.25 |
| DSM-Anxiety |  |  |  |  |  |  |  |  | -0.70 | 0.51 | -.15 | 0.17 |
| Regular psychotropics |  |  |  |  |  |  |  |  | 49 | 17 | .25 | 0.006 |
| ***R*^2^ ** | **0.17** | | | | **0.18** | | | | **0.27** | | | |
| **F for *R^2^*** | F_2,142_=14.1; P<0.001 | | | | F_4,140_=7.6; P<0.001 | | | | F_9,118_=4.8; P<0.001 | | | |
