# Supplementary material for: Conditioned pain modulation identifies altered sensitivity in extremely preterm young adult males and females
Source: Br J Anaesth. 2018 Jul 6;121(3):636–46. doi: 10.1016/j.bja.2018.05.066 (PMC6200113; doi:10.1016/j.bja.2018.05.066)
Supplement: Multimedia component 2 [file mmc2.doc]

**STROBE Statement—Checklist of items that should be included in reports of *cohort studies***

|  | Item No | Recommendation | Page reference and Details  Text taken directly from manuscript highlighted in *italics* |
| --- | --- | --- | --- |
| **Title and abstract** | 1 | (*a*) Indicate the study’s design with a commonly used term in the title or the abstract | Title: *Conditioned Pain Modulation identifies sex-dependent alterations in pain response in extremely preterm born young adults*  Abstract: *This* ***observational cohort study..*** |
|  |  | (*b*) Provide in the abstract an informative and balanced summary of what was done and what was found | Abstract in suggested format: Background, Methods, Results, Conclusions. |
| Introduction | | |  |
| Background/rationale | 2 | Explain the scientific background and rationale for the investigation being reported | Introduction |
| Objectives | 3 | State specific objectives, including any prespecified hypotheses | *In an observational cohort study, we compared CPM in extremely preterm and term born young adults. The primary outcome was identification of modulatory effects (inhibition, facilitation, no change) in EP and TC groups.* |
| Methods | | |  |
| Study design | 4 | Present key elements of study design early in the paper | *observational cohort study* |
| Setting | 5 | Describe the setting, locations, and relevant dates, including periods of recruitment, exposure, follow-up, and data collection | *Participants were recruited from the EPICure cohort of extremely preterm (EP, <26 weeks gestation) and term controls (TC) born in the United Kingdom and Ireland in 1995...*  *The current study at 19 years (EPICure@19; www.epicure.ac.uk)* Following written consent, *participants completed two days of assessments at the NIHR UCLH Clinical Research Facility, London between February 2014 and October 2015.*  *CPM was assessed as part of a comprehensive evaluation of somatosensory function in 102 extremely preterm born and 48 term-born control participants in a dedicated sensory testing facility at UCL GOS Institute of Child Health* |
| Participants | 6 | (*a*) Give the eligibility criteria, and the sources and methods of selection of participants. Describe methods of follow-up | Fig. 1 Recruitment flow chart |
|  |  | (*b*) For matched studies, give matching criteria and number of exposed and unexposed | Figure 1  Table 1 summarises participant demographic data and demonstrates appropriate age and gender match of extreme preterm born (EP, exposed) and term born control (TC, unexposed) groups. |
| Variables | 7 | Clearly define all outcomes, exposures, predictors, potential confounders, and effect modifiers. Give diagnostic criteria, if applicable | Methods include details of outcomes.  Exposures, predictors, potential confounders included in regression model (Table 3) and Supplementary Tables 1, 2, 3. |
| Data sources/ measurement | 8* | For each variable of interest, give sources of data and details of methods of assessment (measurement). Describe comparability of assessment methods if there is more than one group | Assessments included in Methods. Methods of analysis of CPM effect discussed, and both raw and normalized data included.  Validated questionnaire measures. |
| Bias | 9 | Describe any efforts to address potential sources of bias | *Testing was performed by a single investigator (SMW) using standardized verbal instructions in a temperature-controlled room at the same time of day.* |
| Study size | 10 | Explain how the study size was arrived at | Recruitment discussed. 95%CI rather than post-hoc power analysis to support power. |
| Quantitative variables | 11 | Explain how quantitative variables were handled in the analyses. If applicable, describe which groupings were chosen and why | Statistical analysis section |
| Statistical methods | 12 | (*a*) Describe all statistical methods, including those used to control for confounding | Statistical Analyses section in Methods. Additional test in Results, Figure Legends, and Tables. |
|  |  | (*b*) Describe any methods used to examine subgroups and interactions | Table 2 Correlation matcrix and Table 3 Regression model; Supplementary Tables 1and 2: correlation between variables separated by preterm versus control groups, and by conditioning stimulus tolerance. |
|  |  | (*c*) Explain how missing data were addressed | Tables: Data cells with missing data contain actual ‘n’ for available data. No imputation for missing data was performed. |
|  |  | (*d*) If applicable, explain how loss to follow-up was addressed | EP participants evaluated at 19 years did not differ in birth weight, gestational age or sex from those lost to follow-up, but had higher socio-economic status and higher mean IQ scores at earlier assessments than non-participants. |
|  |  | (*e*) Describe any sensitivity analyses |  |
| Results | | |  |
| Participants | 13* | (a) Report numbers of individuals at each stage of study—eg numbers potentially eligible, examined for eligibility, confirmed eligible, included in the study, completing follow-up, and analysed | Figure 1 |
|  |  | (b) Give reasons for non-participation at each stage | Figure 1 |
|  |  | (c) Consider use of a flow diagram | Figure 1 is presented as a flow diagram of participant numbers at each stage of the study. |
| Descriptive data | 14* | (a) Give characteristics of study participants (eg demographic, clinical, social) and information on exposures and potential confounders | Table 1 |
|  |  | (b) Indicate number of participants with missing data for each variable of interest | Tables include sample size (n=) at head of column. Data cells with missing data contain actual ‘n’ for available data. No imputation for missing data was performed. |
|  |  | (c) Summarise follow-up time (eg, average and total amount) |  |
| Outcome data | 15* | Report numbers of outcome events or summary measures over time | Recruitment and outcome at different stages of this longitudinal study have been previously reported and are summarised here.  1. Wood NS et al. *N Engl J Med.* 2000;343(6):378-384.  2. Marlow N et al. *N Engl J Med.* 2005;352(1):9-19.  3. Johnson S et al. *Pediatrics.* 2009;124(2):e249-257. |
| Main results | 16 | (*a*) Give unadjusted estimates and, if applicable, confounder-adjusted estimates and their precision (eg, 95% confidence interval). Make clear which confounders were adjusted for and why they were included | In tables, data are presented as mean±SD if normally distributed or median and interquartile range. Comparisons and graphs include individual data points and/or mean [95%CI]. |
|  |  | (*b*) Report category boundaries when continuous variables were categorized | Graphs include individual data points and/or mean [95%CI]. |
|  |  | (*c*) If relevant, consider translating estimates of relative risk into absolute risk for a meaningful time period | N/A |
| Other analyses | 17 | Report other analyses done—eg analyses of subgroups and interactions, and sensitivity analyses |  |
| Discussion | | |  |
| Key results | 18 | Summarise key results with reference to study objectives | Discussion |
| Limitations | 19 | Discuss limitations of the study, taking into account sources of potential bias or imprecision. Discuss both direction and magnitude of any potential bias | Paragraph in Discussion reports Limitations. |
| Interpretation | 20 | Give a cautious overall interpretation of results considering objectives, limitations, multiplicity of analyses, results from similar studies, and other relevant evidence | Final paragraph of Discussion: Summary |
| Generalisability | 21 | Discuss the generalisability (external validity) of the study results | Included in Discussion |
| Other information | | |  |
| Funding | 22 | Give the source of funding and the role of the funders for the present study and, if applicable, for the original study on which the present article is based | Funding and Acknowledgements sections included. |

*Give information separately for exposed and unexposed groups.

**Note:** An Explanation and Elaboration article discusses each checklist item and gives methodological background and published examples of transparent reporting. The STROBE checklist is best used in conjunction with this article (freely available on the Web sites of PLoS Medicine at http://www.plosmedicine.org/, Annals of Internal Medicine at http://www.annals.org/, and Epidemiology at http://www.epidem.com/). Information on the STROBE Initiative is available at http://www.strobe-statement.org.
